# Efficacy of a new treatment algorithm for capsulitis of the fingers in rock climbers

**DOI:** 10.3389/fspor.2025.1497110

**Published:** 2025-01-20

**Authors:** Volker Rainer Schöffl, Christoph Lutter, Hans-Christoph Lang, Mario Perl, Othmar Moser, Michael Simon

**Affiliations:** ^1^ Department of Trauma and Orthopedic Surgery, Klinikum Bamberg, Bamberg, Germany; ^2^ Department of Trauma and Orthopedic Surgery, Friedrich-Alexander University Erlangen-Nuremberg, University Hospital Erlangen, Erlangen, Germany; ^3^ Section of Wilderness Medicine, Department of Emergency Medicine, University of Colorado School of Med., Denver, CO, United States; ^4^ School of Health, Leeds Becket University, Leeds, United Kingdom; ^5^ Division of Exercise Physiology and Metabolism, Department of Sport Science, University of Bayreuth, Bavaria, Germany; ^6^ Department of Orthopedics, University Medical Center, Rostock, Germany

**Keywords:** capsulitis, finger joint synovitis, rock climbing, finger injuries, sport climbing

## Abstract

**Background:**

Although finger joint capsulitis has been described among the most frequent injuries in climbers, no clinical studies on treatment strategies and outcomes are available.

**Study design:**

Prospective case series study.

**Methods:**

Between 2015 and 2018 we prospectively treated 50 patients (38 male, 12 female) with a total number of 69 independent finger joint capsulitis according to a clinic specific treatment regimen and evaluated the outcome retrospectively. Therapy consisted of either conservative management, steroid injections, radiosynoviorthesis or a combination depending on the treatment regimen, prior therapy and timeline of symptoms. Outcomes were assessed using visual analogue scale (VAS), Buck-Gramcko score and a climbing specific outcome score with secondary patient recall.

**Results:**

The proximal interphalangeal joint of the middle finger was the most commonly affected joint, and there was no correlation with osteoarthritis. All climbers returned to sport within 12 months. The majority were able to maintain their level of performance after injury and the difference in climbing level before and after injury was not statistically significant (*p* = 0.22). The total time spent climbing was significantly less after the injury than before the injury (*p* < 0.001). The Buck-Gramcko score showed excellent results. The overall functional outcome was good to very good with a mean score of 1.6 ± 0.7, as was the climbing specific score of 1.7 ± 0.9. Pain was significantly less after treatment than before (*p* < 0.001).

**Conclusion:**

Good to very good functional and sport-specific outcomes were seen with the stage-specific treatment regimen presented, allowing all patients studied to resume climbing. A better understanding of the underlying pathogenesis is essential in order to better assess long-term progress.

## Introduction

Finger injuries are the most common sport-specific injuries in rock climbing (1–3). With the growing popularity of the sport, which is an Olympic sport since the 2020 summer games in Tokyo (held in 2021), and the ever-increasing difficulty of the sport, we can expect to see an increase in training intensity and chronic overuse injuries (1, 2, 4–7). After finger flexor pulley lesions and tenosynovitis of the flexor tendons, chronic capsulitis of the small finger joints was found to be the third most common diagnosis seen in climbers (7.7% in overall 633 injured climbers) (8). In an analysis of the differential diagnosis of finger injuries, it was reflected in 18.8% of 261 finger injuries (8). Up to date now work exists into further analyzing this condition, its pathogenesis, treatment possibilities and outcome analysis.

**Figure 1:**
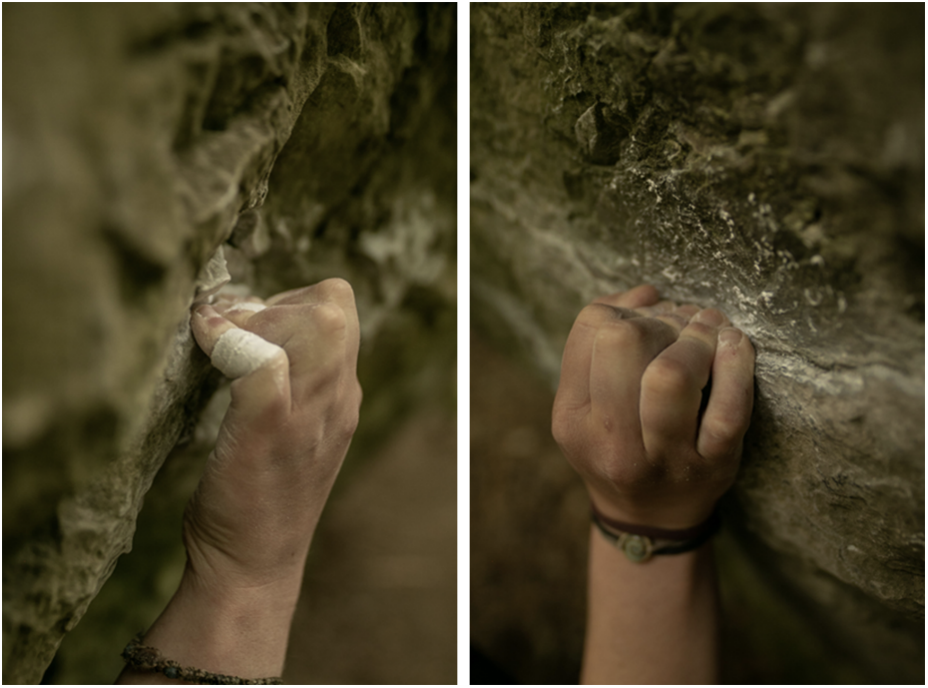
Crimp grip during rock climbing. The crimp grip with an angle between 90° and 110° in the PIP joint is preferred by climbers because, among other reasons, the flexor tendons of the fingers can develop the greatest possible holding force due to the resulting lever arm.

The term capsulitis (intra-articular synovitis) has been used in literature on climbing injuries to describe a variety or complex of symptoms consisting of hyperemia and effusion of the finger joints in climbers (8–11). Despite being one of the most common finger injuries in climbers (1, 12) neither the etiology nor the treatment has been extensively analyzed so far (10, 13). In terms of etiology, the following theory is currently being discussed as the most likely: High peak pressures within the interphalangeal joints of the fingers, especially during the crimp position (Figure 1), cause the release of inflammatory mediators that can trigger chronic inflammation of the joint capsule (8). Capsulitis can therefore be caused either by repetitive stress or by a single traumatic event with reactive effusion within the joint (8). This effusion itself affects the synovia and can be the starting point of a vicious cycle of overstrain, effusion and synovitis, leading to a chronic capsulitis and, if left untreated, potentially to osteoarthritis and joint destruction (8, 9, 13–15); however, this theory has yet to be scientifically proven. It is debatable whether the term “synovitis” would not be more appropriate, but the term “capsulitis” has become popular in the climbing literature (8, 11). This can also be compared to the foot surgeons using the term “dactylitis” for synovitis of the joints of the small toes. We therefore decided to use the term “capsulitis” in this paper.

**Figure 2:**
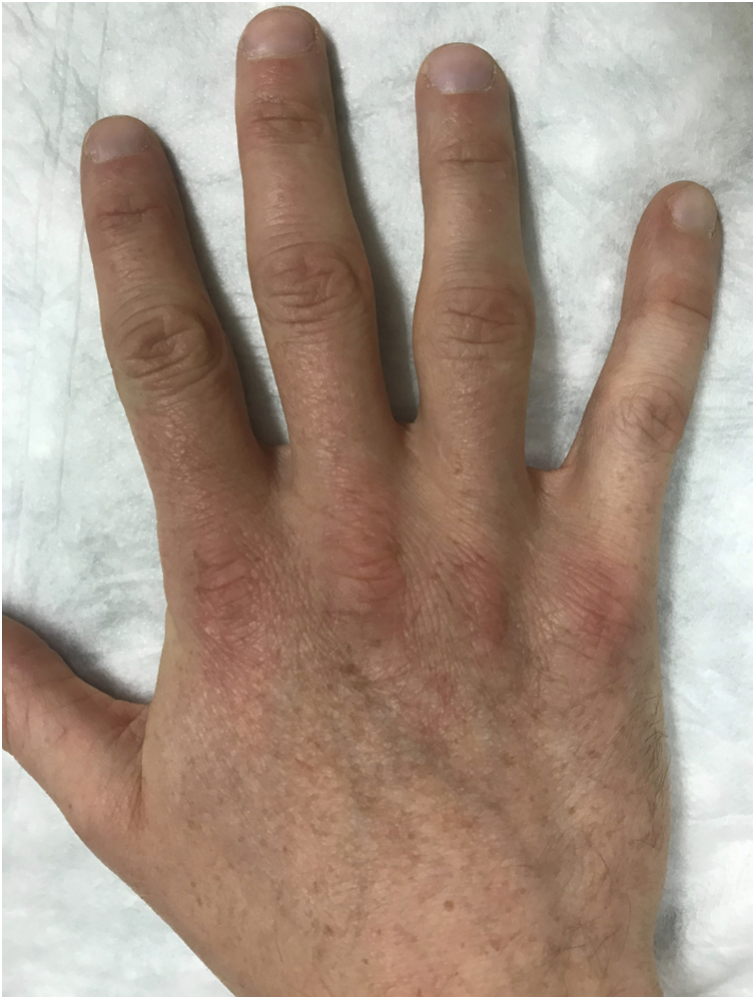
Clinical presentation of a patient's finger hand with capsulitis on the right ring finger proximal interphalangeal (PIP) joint.

**Figure 3:**
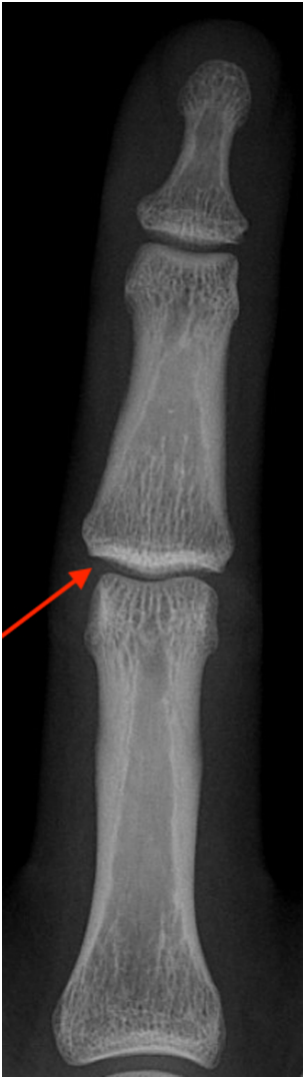
X-ray of a capsulitis of the PIP joint depicting as a joint space widening due to the effusion (“x-ray sign 1”, arrow) as well as a thickened soft tissue presentation (“x-ray sign 2”) (9).

Most climbers complain of early morning stiffness and swelling in the affected finger joints, reduced range of motion and pain, which often improves after activity (Figure 2) (8, 10, 11, 13). Clinical findings include swollen finger joints and dorsal pain to tenderness and palpation of the affected joint. The diagnosis can be confirmed by ultrasound, which shows effusion and an increased synovial blood flow (10, 16–18). In addition, radiographs may show joint space widening (“x-ray sign 1”, Figure 3, red arrow) and soft tissue thickening due to a swollen inflammatory edematous synovial membrane (“x-ray sign 2”, Figure 3) (9). In addition, the diagnosis may be confirmed by magnetic resonance imaging using an intravenous contrast agent (e.g., gadolinium) (2, 15). There are several experience-based treatment options for capsulitis in climbers: besides conservative options such as rest, icing, anti-inflammatory drugs (NSAID'S) and anti-rheumatic drugs (e.g., methotrexate), intra-articular injections of platelet rich plasma (PRP), hyaluronic acid or steroids and radiosynoviorthesis (using 20MBq Erbium) are therapeutic options (8–11, 13–15, 19). As a last resort, local radiotherapy or a surgical synovectomy may be considered (10). To standardize management we developed a stage-related treatment regimen for capsulitis of the finger joints in climbers (Table 1) based on the evaluation and management of affected climbers (not included in the current study sample) presenting to our clinic, which is a referral center for climbing injuries (8).

**Table 1:** Stage-related treatment regimen for capsulitis of the finger joints in climbers (8).

Stage	Time frame since onset of pain	Therapy	Climbing rest
1	<6 weeks	Conservative: icing, movement therapy, traction, hand baths with anti-inflammatory solution (e.g., medical sulphur solution), exercise therapy with therapy putty, acupressure ring, lymphatic drainage.	• 0–14 days • Then stepwise climbing load increase in accordance to the clinical symptoms. • Taping during climbing restart with figure of eight tape.
2	>6 weeks (or after failed conservative therapy >4 weeks)	2a. local infiltration with corticosteroid, re-injection after 7–10 days. 2b. alternative local infiltration with PRP (Platelet-rich plasma), mesenchymal stem cells (MSC) or autologous fat micrograft. (If applicable combination of corticosteroid or PRP).	• 2a: no climbing or hand related sports in between the injections and at least for 10 days after the second injection. • 2b*:* no climbing or hand related sports 48 h after the injection. • Then stepwise climbing load increase. • Taping during climbing restart with figure of eight tape.
3	Persistent pain and swelling/effusion >6 weeks after second injection	RSO (Radiosynoviorthesis using 20MBq Erbium).	• Immobilization of the respective joint for 48 h on a splint. • Afterwards light movement exercises; gradually restart climbing activities after 6 weeks, with full load bearing after 10 weeks. • Taping during climbing restart with figure of eight tape.
4	Persistent pain and swelling/effusion >6 month after first RSO (20MBq Erbium)	Second RSO if applicable in combination with simultaneous instillation of a corticoid or Medical leech therapy or Local radiation therapy (last resort: surgical synovectomy)	• Immobilization of the respective joint for 48 h on a splint. • 6 weeks rest, then stepwise climbing load increase with full load bearing after 10 weeks. • Taping during climbing restart with figure of eight tape. • Personalized follow-up regime in case of medicinal leech therapy, radiation, or synovectomy.

The aim of this study was to investigate the treatment success (clinical and sport-specific outcome) of climbers treated for finger joint capsulitis using a staged treatment regimen.

## Methods

### Patient criteria

Between 2015 and 2018, all climbers (non-competitive and competitive, indoor and outdoor) who were treated for capsulitis of the proximal (PIP) or distal (DIP) interphalangeal joint or the metacarpophalangeal joint (MCP) of the finger were included in this study. The climbers were seen and treated at our specialized outpatient sports medicine clinic, which is a referral center for climbing-related injuries (e.g., German Alpine Club). All 50 patients [38 (67%) male, 12 (24%) female] with a total number of 69 independent finger joint capsulitis injuries included in the study complained of pain for more than 6 weeks during or after climbing. The mean age at presentation was 35.6 ± 10.1 years, and the mean BMI was 22.2 ± 2.6 (Table 2). Climbing and bouldering skill levels and demographic information of the patient population are shown in Table 2. A climbing-specific score was used to assess the pre-injury status of the affected finger (Table 3) (20, 21). The study was approved by the institutional ethics committee and all patients provided informed consent.

**Table 2:** Patient characteristics.

	All patients
Number of patients	50
Number of injuries	69
Age (years)	35.6 ± 10.1
Gender (men/women)	38/12
BMI	22.2 ± 2.6
Dominant hand r/l	48/2
Climbing sport experience	12.5 ± 8.5 years
Rope climbing level° before injury	8.6 ± 1.1
Rope climbing level° after therapy	8.3 ± 1.8
Bouldering V-scale before injury	7.9 ± 0.9
Bouldering V-scale after therapy	7.8 ± 1.0
Recreational climber	42 (84)
Competitive climber	7 (14)
Professional climber	1 (2)
Training:	
1–2 sessions/week	6 (12)
2–3 sessions/week	24 (48)
3–5 sessions/week	19 (38)
>5 sessions/week	1 (2)
Conservative treatment	26 fingers
Steroid injections	38 fingers
Steroid injections + RSO	5 fingers

UIAA metric grade.

### Diagnostics

A standard questionnaire and examination protocol were used. Diagnosis was based on clinical investigation and radiologic findings. The final diagnosis was reviewed and confirmed by the first author, who is a board-certified orthopedic surgeon with more than 25 years of experience with climbing injuries. Patients who were initially seen and treated in our emergency department were later re-evaluated in the outpatient sports medicine clinic.

All patients presenting to our center with a diagnosis of proximal interphalangeal (PIP), distal interphalangeal (DIP) or metacarpophalangeal (MCP joint capsulitis were included. Patients were excluded if they had finger pain, osteoarthritis, rheumatoid or psoriatic arthritis prior to rock climbing or any underlying relevant medical condition or hand surgery. Capsulitis was defined as: swelling, pain and tenderness of the respective joint with effusion (PIP, DIP) for more than 6 weeks; pain and swelling aggravated by climbing; limited range of motion; absence of chronic osteoarthritis; joint effusion and possible hyperemia in the capsule (ultrasound and/or MRI); absence of pulley or collateral ligament injury, joint capsular sprain or any other climbing finger-specific differential diagnosis (10).

### Classification

The *Union Internationale des Associations d'Alpinisme* (UIAA) metric scale was used to grade climbing levels and the V scale (Vermin scale) was used to grade bouldering levels as previously published (12, 22). The (UIAA) Injury Score was used to classify the severity of injury, as recommended by the UIAA Medical Commission (22).

### Therapeutic regimen

The clinic-specific, experience-based, stage-related treatment regimen for capsulitis of the finger joints in climbers (Table 1) was employed for all patients involved in this study. Conservative therapy consisted of icing, movement therapy, physiotherapy with traction of the PIP joint, hand baths with anti-inflammatory solution (e.g., medical sulfur solution), exercise therapy with theraeuticy putty, acupressure ring, lymphatic drainage and stress reduction (climbing rest).

### Outcome

Climbers were seen for follow-up at 6 and 12 weeks after the initial visit and contacted at 1 year. Visual Analog Scale (VAS) scores were collected at the initial visit and at one year. In addition, the Buck-Gramcko score a functional outcome score after finger injuries in climbing and a climbing-specific outcome score (Tables 3A,B) were recorded at 1 year (20, 21, 23). The Buck-Gramcko is a common hand surgery score for the outcome analysis of flexor tendon injuries focusing on range of motion and pain (23). It has been widely used in outcome analysis of finger injuries of climbers (24–28). All patients self-reported pre-injury conditions. Patients' self-assessments of time to treatment success (pain reduction, swelling reduction, increased range of motion, increased climbing) were then evaluated.

**Table 3A:** Functional outcome score after finger injuries in climbing according to Schöffl et al. (20, 21).

Excellent (1)	Free range of motion, no objective strength deficit, normal motion pattern.
Good (2)	Extension- or flexion deficit of the PIP joint up to 10°, minor strength deficit, normal motion pattern.
Satisfactory (3)	Extension- or flexion deficit of the PIP joint up to 20°, clinical strength deficit, minor irritation of motion pattern.
Fair (4)	Extension- or flexion deficit of the PIP joint of more than 20°, major strength deficit, major irritation of motion pattern.

(PIP, proximal interphalangeal joint).

**Table 3B:** General outcome score for climbing injuries according to Schöffl et al. (20, 21).

Excellent (1)	Full load capacity of the former injured joint/extremity after 12 months (with or without tape), no subjective strength deficit of the former injured joint, regain of full climbing ability (UIAA metric +/− 0.66 UIAA metric grade)/pre-injury climbing level, no pain.
Good (2)	**F**ull load capacity of the former injured joint/extremity after 12 months (with tape), subjectively minor strength deficit of the former injured joint/extremity, regain of full climbing ability (UIAA metric +/− 0.66 UIAA metric grade)/pre-injury climbing level, minor pain.
Satisfactory (3)	Minor restricted load capacity of the former injured joint/extremity after 12 months (with tape), subjectively strength deficit of the former injured f joint/extremity, regain of full climbing ability/pre-injury UIAA climbing level minus one UIAA metric grade, minor pain.
Fair (4)	Major restricted load capacity of the former injured joint/extremity after 12 months (with tape), strength deficit und restricted ability to use the former injured finger while climbing, major decrease in climbing ability (more than 1.33 UIAA metric grade), frequent pain.
Poor (5)	Climbing is not possible anymore.

### Statistical analysis

Microsoft Excel (Microsoft, Redmond, Washington, USA) was used for data collection Statistical analysis was performed using GNU PSPP Statistical Analysis Software (Free Software Foundation Inc. 2007, version 1.4.1-g79ad47, Boston, Massachusetts, USA). The Kolmogorov-Smirnow test was used to test for normal distribution. *T*-test was used for normal distributed data, Wilcoxon rank test was used for not normal distributed data. Level of significance was set as *p* < 0.05. Unless otherwise noted, data was expressed as mean ± SD (standard deviation).

## Results

Thirty-seven patients (37/50) presented with one affected finger, 8 patients with two, 4 patients with three, and one patient with four individual finger injuries. The clinical characteristics are shown in Table 4. In 38 cases (55%), patients reported a chronic onset of symptoms; in 31 (45%), an acute onset of symptoms was reported during or a few days after a climbing session. Four patients reported a specific event with immediate onset of symptoms: in three cases, pain occurred after holding a one-finger pocket, while one climber reported trapping his finger in a finger crack. Climbers reported diffuse pain with lateral and dorsal pressure tenderness in the affected joint with intensification during flexion and extension and during and after climbing, proportional to the intensity of training. The time interval between the onset of finger joint symptoms and the first consultation was 26.7 ± 40.1 weeks (range: 3 days to 2.5 years). None of the climbers reported any history of the affected fingers, and all had excellent pre-injury climbing scores.

**Table 4:** Clinical characteristics.

	All patients
Number of patients	50
Number of injuries	69
Affected finger	
Dig III right	24 (34.8)
Dig III left	18 (26.1)
Dig IV left	11 (15.9)
Dig IV right	6 (8.7)
Dig II left	5 (7.2)
Dig II right	4 (5.8)
Dig V left	1 (1.5)
Affected joint	
PIP joint	57 (82.6%)
DIP joint	8 (10.2%)
MCP joint	4 (5.8%)
Clinical symptoms	
Pain	64 (92.8%)
Joint swelling	31 (44.9%)
Restriction of movement	9 (13%)
Morning stiffness	7 (10.1%)
Joint hyperthermia	2 (2.9%)
Joint snapping	2 (2.9%)
Misalignment of joint	1 (1.5%)
Onset of symptoms	
Acute	31 (44.9%)
Chronic	38 (55%)
Osteoarthritis^a^	
Grade 0	43 (62.3%)
Grade 1	24 (34.8%)
Grade 2	1 (1.5%)
Grade 3	1 (1.5%)
Grade 4	0

^a^ Stages of arthritic changes in imaging according to Kellgren and Lawrence score (29).

According to the treatment algorithm, 26 fingers were treated conservatively and 38 were treated with steroid injections. Five fingers required additional treatment with radiosynoviorthesis (RSO, using erbium) after cortisone injection. None of the patients required extended treatment (stage 4).

### Return to sport

The interval from the end of therapy (in case of conservative treatment after the last consultation, in case of cortisone injection and RSO after the last therapy session) to the resumption of (climbing) sports activity was 3.7 ± 4.5 weeks (range 0–30 weeks). After conservative therapy, return to sport took slightly longer (4.3 ± 6.7 weeks) than after steroid injection (3.4 ± 2.3 weeks) and steroid + RSO (2.75 ± 1 weeks). At 12.6 ± 12.6 weeks after treatment, 93% of all treated fingers had regained full weight bearing. In 5 fingers (3× conservatively treated, 1× cortisone injection, 1× RSO), full weight bearing was not yet possible at the time of reevaluation. Twenty climbers still had mild pain (VAS 2.2 ± 1.4) with certain movements and loads at the time of the interview, and 30 climbers were pain-free. Pain was significantly less after treatment than before (*p* < 0.001).

There was a significant change in total climbing time from pre-injury to post-treatment (*p* < 0.001). While 86% of the climbers climbed 2–4 times per week before the injury, this number decreased to 66% after the injury. In terms of the cause of the reduction in climbing time, it was not due to the injury or its aftermath; the patients reported that they had rearranged their leisure activities during rehabilitation.

### Outcomes

**Figure 4:**
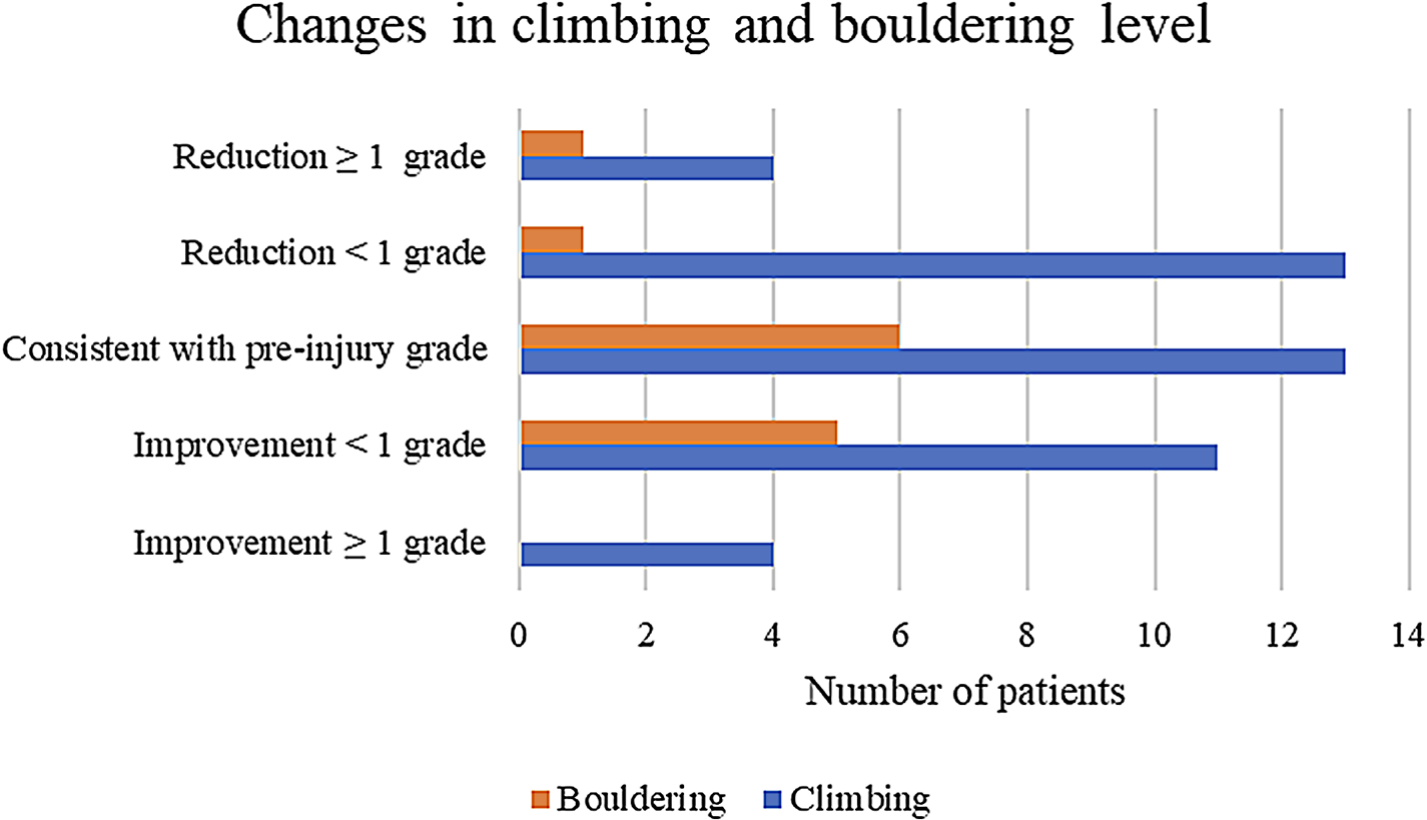
Changes in climbing and bouldering levels of affected climbers. The majority of climbers regained their pre-injury climbing and/or bouldering level; nineteen climbers reported a reduction of their climbing performance: <1 climbing/boulder grade less: *n* = 14, reduced climbing performance of ≥1 grade: *n* = 5. The difference of the climbing level of pre-injury to post-injury was not significant (*p* = 0.22).

All climbers had returned to sport within 12 months. The majority (72.4%) were able to maintain their performance level after the injury (Figure 4), and the difference of climbing level before and after injury was not statistically significant (*p* = 0.22).

The range of motion of the individual fingers at the time of the examination showed a Buck-Gramcko score of 14.3 ± 1.3, which represents excellent results (Buck-Gramcko scores: 49× 15, 1× 14, 13× 13, 4× 11, 2× 10). Twenty-one of the 69 (30.4%) fingers showed minor motion restrictions; two of these fingers (6.8%) were restricted in both flexion and extension: 18× flexion deficit (16.4° ± 4.5°), 5× extension deficit of 12° ± 5.7°. The exact distribution is shown in Table 5. The overall functional outcome was good to very good with an average score of 1.6 ± 0.7 (functional outcome score after finger injuries in climbing), and an climbing specific score of 1.7 ± 0.9. Seventy-five percent of the participating climbers indicated that they were satisfied with the duration of the healing process, while 25% considered the healing time to be inconveniently long. Outcome scores separated by treatment regimen are shown in Table 6.

**Table 5:** Range of motion outcomes after therapy.

Restriction in range of motion		Affected fingers/total		%	Restriction in°
Overall		21/69		30.4	15.4° ± 5.6° (10–30)
Flexion deficit	Finger	Dig III	13/42	30.1	16.4**°** ± 4.5**°** (10–30)
		Dig II	3/9	33.3	
		Dig IV	2/17	11.8	
	Joint	PIP	15/58	25.9	
		MCP	2/4	50	
		DIP	1/7	14.3	
Extension deficit	Finger	Dig III	3/42	7.1	12**°** ± 5.7**°** (10–20)
		Dig II	2/9	22.2	
	Joint	PIP	5/58	8.6	

**Table 6:** Outcome scores separated by treatment regimen.

		Conservative *n* (%)	Cortisone *n* (%)	Cortisone + RSO* *n* (%)	Total *n* (%)
Overall functional outcome	1	16 (62)	20 (53)	1 (20)	37 (54)
	2	10 (38)	12 (32)	1 (20)	23 (33)
	3		6 (16)	3 (60)	9 (13)
	4				
		1.4	1.6	2.4	1.6
Sport/climbing specific outcome	1	15 (58)	21 (55)	1 (20)	37 (54)
	2	10 (38)	9 (24)	2 (40)	21 (30)
	3	1 (4)	5 (13)	2 (40)	8 (12)
	4	–	3 (8)	–	3 (4)
	5	–	–	–	–
		1.5	1.7	2.2	1.7

* RSO, radiosynoviorthesis.

## Discussion

This is the first study to evaluate the results of a structured treatment regimen for the treatment of climbing related finger joint capsulitis. A total of 50 patients with capsulitis of one or more fingers (69 in total) were treated and evaluated with good to very good results in both general functional and sport-specific terms.

With increasing invasiveness of the treatment (conservative vs. injection therapy vs. RSO), the therapeutic outcome slightly worsened. However, a direct comparison of the three different treatment modalities based on this study is not possible because the treatment was selected sequentially according to the treatment algorithm. Finger mobility remained slightly limited in 30.4% (21 fingers) of the cases. This did not lead to any impairment in daily life. However, 19 climbers (28%) experienced a decrease in climbing and bouldering difficulty compared to the pre-injury period (Table 1; Figure 4). In addition to residual symptoms that persisted despite therapy, this may be due to the sometimes long interval between symptom onset and full recovery after therapy and the resulting reduced training workload. Furthermore, in almost all cases the reduction in climbing frequency was not due to the capsulitis; some patients reported that they used the injury and the resulting long forced break as an opportunity to redirect their leisure activities. A decrease in climbing ability after injury was lately also reported about in finger tenosynovitis (30), wrist injuries (31), as well as shoulder injuries [rotator cuff tears (32), SLAP lesions (33) and dislocations (34)].

The injury susceptibility of the different fingers in the current study correlates with the relevant biomechanics literature (35–37). As the longest finger, the middle finger absorbs the most force, experiences the most stress and is therefore the most prone to injury, which is also reflected in the results of our study (38). This is followed by the ring finger, index finger and little finger (10). No capsulitis was diagnosed in the thumb, as this finger is only used for active support during climbing and (unlike fingers II–V) is never subject to a load similar to that of the long fingers (37). Looking at the different grip shapes used in climbing, similar to the correlation between finger flexor pulley injuries and the crimp grip (Figure 1) (39), an association with the crimp grip is also likely for the development of capsulitis, which is reflected in the strikingly frequent anamnestic indication of capsulitis-associated pain when holding ledges with this grip shape. The crimp grip with an angle between 90° and 110° in the PIP joint is preferred by climbers because, among other reasons, the flexor tendons of the fingers can develop the greatest possible holding force due to the resulting lever arm (40, 41). Studies have shown that the crimp grip places high stress on the cartilage of the finger joints (35, 39, 42–44).

**Figure 5:**
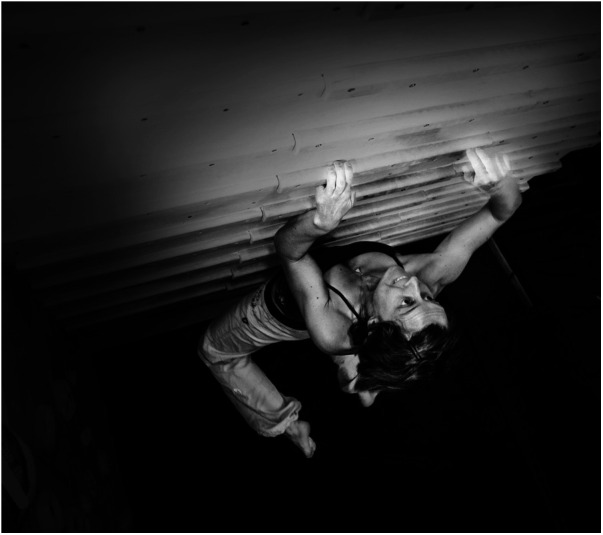
Campus board training: a campus board is a specific training device for climbers and consists of very narrow horizontal holds on an inclined vertical board. For ascending only the hands are used.

With the increasing number of patients presenting with clinical signs of capsulitis, especially those who have been climbing for a short time, the question of possible preventive measures arises (1, 2, 4–6, 45). This requires a slow adaptation of the musculoskeletal system to the increased load and education of the climbers about potential overuse injuries. For example, prevention programs should be implemented to avoid premature overuse of the small finger joints through overly intensive training, such as hanging from the smallest ledges during campus board training (Figure 5).

The definitive question of the etiology of climbing-related capsulitis and whether it is a form of pre-arthritis remains unclear. While it is known that the crimp grip places high stress on the cartilage of the finger joints it is still unclear in which cases it causes synovial inflammation in the finger joints (35, 39, 42–44). Histologic studies of the knee joint have shown a direct correlation between arthritic joint symptoms and the presence of synovial inflammation, with the amount of symptoms correlating with the degree of synovial inflammation (14). In addition, patients with clinical symptoms but no radiographic evidence of osteoarthritis may be diagnosed with synovial inflammation (14, 46). Capsulitis was diagnosed clinically, sonographically, and radiographically in the climbers in this study, with no radiographic evidence of osteoarthritis in the majority of patients (62%). Definitive molecular detection of synovitis by biopsy is not feasible in the small finger joints of climbers due to the highly invasive nature of the procedure, nor does it provide any therapeutic benefit. If the common models for the development of synovial inflammation from studies primarily on the knee joint are applied to the small finger joints of climbers, a multifactorial genesis of capsulitis can be assumed, independent of the presence of osteoarthritis. Therefore, the following model serves as a possible explanation: Acute trauma, but especially chronic climbing-induced micro-trauma of the small finger joints could trigger an aseptic immune response via the release of damage-associated molecular patterns (DAMPs), activation of the complement system and activation of proinflammatory cytokine cascades mediated by specific mechanoreceptors (14, 46–48). Chondrocytes can be stimulated by specific mechanical stimuli to produce inflammatory mediators and trigger the catabolic activity of macrophages (49). Intensive, both acute and chronic stress causing increased expression of proinflammatory mediators in studies whereby NF-kB (*nuclear factor* “*kappa-light-chain-enhancer*” *of activated B-cells*) appears to be an important transcription factor (50, 51). Its activity is suppressed by intermittent, cyclic mechanical loading of chondrocytes, but is stimulated by high-intensity, static loading (52). The finger joints perform mainly static holding work during climbing, which supports this explanation. Other theories for the development of capsulitis, such as pre-existing cartilage or bone degradation as in detritus synovialitis, in which cartilage or bone fragments in the joint space initiate the inflammatory response of the synovium seem less likely (53). However, this is contradicted by the high proportion of patients in the current study who had no radiographic signs of osteoarthritis. In addition, climbers with radiographic evidence of osteoarthritis, such as osteophyte formation, may not have symptoms typical of capsulitis. An example of this is a professional climber in the study group who developed symptomatic capsulitis based on decades of asymptomatic, radiographic finger joint osteoarthritis (Kellgren-Lawrence grade 2). Also, Hochholzer et al. (54) as well as Lutter et al. (55) report about many cases of elderly climbers with radiographic osteoarthritis but no evidence of acute swelling or capsulitis. Further studies are needed to investigate and validate our hypothesized explanatory model.

The use of advanced magnetic resonance imaging and molecular imaging as a non-invasive diagnostic tools to better understand the metabolic processes in patients with capsulitis without performing a biopsy is promising (56). Long-term studies are needed to clarify to what extent capsulitis may be an early form of osteoarthritis without radiologic correlates, whether these patients with persistent symptoms and prolonged climbing-specific stress develop manifest osteoarthritis, and the role of correct diagnosis and stage-appropriate therapeutic intervention.

### Limitations

This study is not without limitations, as a comparative evaluation of this treatment regimen cannot be made due to the lack of studies on climbing specific capsulitis. In addition, there is no comparison with a control group to be able to better classify the very positive treatment results. A control group with no treatment would have been scientifically advantageous, but difficult to ask from our patients. It is well known in climbing medicine that capsulitis is a long-term and often chronic condition with little self-healing capacity (8, 10). Furthermore, in none of the patients was it necessary to extend the treatment to stage 4 of our treatment algorithm (Table 1), so these treatment options (second RSO with simultaneous instillation of a corticoid, medical leech therapy, local radiation therapy, surgical synovectomy) were not evaluated. Further studies are needed to investigate these potentially promising treatment options and compare them with the previous results. The relatively small number of cases in our study should also be noted. This is because sports-related capsulitis of the small finger joints is almost exclusively found in climbing. Also, a direct comparison of the three different forms of treatment modalities is not possible in this study as the treatment was selected sequentially according to the treatment algorithm. In addition, there is a lack of knowledge in the area of conservative treatment, such as techniques, their order or combination, frequency and intensity.

## Conclusion

Although finger joint capsulitis has been described as one of the most common injuries in climbers, this is the first study on the treatment of this injury in a larger cohort. Good to very good functional and sport-specific outcomes were achieved with a stage-specific treatment regimen that allowed all patients to return to climbing. A better understanding of the underlying pathogenesis is essential to better assess long-term outcome.

## Data Availability

The raw data supporting the conclusions of this article will be made available by the authors, without undue reservation.
